# Serum adipocyte fatty acid-binding protein levels in patients with critical illness are associated with insulin resistance and predict mortality

**DOI:** 10.1186/cc12498

**Published:** 2013-02-01

**Authors:** Chi-Lun Huang, Yen-Wen Wu, Ai-Ru Hsieh, Yu-Hsuan Hung, Wen-Jone Chen, Wei-Shiung Yang

**Affiliations:** 1 Department of Internal Medicine, Taoyuan General Hospital, 1492 Chung-Shan Rd., Taoyuan City 33004, Taiwan; 2 Department of Internal Medicine, National Taiwan University Hospital, 7 Chung Shan S. Rd., Taipei City 10002, Taiwan; 3 Graduate Institute of Clinical Medicine, College of Medicine, National Taiwan University, 7 Chung Shan S. Rd., Taipei City 10002, Taiwan; 4 Department of Nuclear Medicine, National Taiwan University Hospital, 7 Chung Shan S. Rd., Taipei City 10002, Taiwan; 5 Department of Nuclear Medicine and Cardiovascular Medical Center (Cardiology), Far Eastern Memorial Hospital, 21 Nanya S. Rd. Sec. 2, New Taipei City 22060, Taiwan; 6 National Yang-Ming University School of Medicine, 155 Li-Nong St. Sec. 2, Taipei City 11221, Taiwan; 7 Institute of Biomedical Science, Academia Sinica, 128 Academia Rd. Sec. 2, Taipei City 11529, Taiwan; 8 Department of Emergency Medicine, National Taiwan University Hospital, 7 Chung Shan S. Rd., Taipei City 10002, Taiwan

## Abstract

**Introduction:**

Hyperglycemia and insulin resistance are commonplace in critical illness, especially in patients with sepsis. Recently, several hormones secreted by adipose tissue have been determined to be involved in overall insulin sensitivity in metabolic syndrome-related conditions, including adipocyte fatty-acid binding protein (A-FABP). However, little is known about their roles in critical illness. On the other hand, there is evidence that several adipose tissue gene expressions change in critically ill patients.

**Methods:**

A total of 120 patients (72 with sepsis, 48 without sepsis) were studied prospectively on admission to a medical ICU and compared with 45 healthy volunteers as controls. Various laboratory parameters and metabolic and inflammatory profiles were assessed within 48 hours after admission. Clinical data were collected from medical records.

**Results:**

Compared with healthy controls, serum A-FABP concentrations were higher in all critically ill patients, and there was a trend of higher A-FABP in patients with sepsis. In multivariate correlation analysis in all critically ill patients, the serum A-FABP concentrations were independently related to serum creatinine, fasting plasma glucose, total cholesterol, TNF-alpha, albumin, and the Acute Physiology and Chronic Health Evaluation II scores. In survival analysis, higher A-FABP levels (> 40 ng/ml) were associated with an unfavorable overall survival outcome, especially in sepsis patients.

**Conclusions:**

Critically ill patients have higher serum A-FABP concentrations. Moreover, A-FABP may potentially serve as a prognostic biomarker in critically ill patients with sepsis.

## Introduction

Intensive stress responses are common in critically ill patients who face life-threatening situations. These responses include inflammatory and metabolic components. They activate central nervous system and neuroendocrine axes, increasing the release of cortisol, epinephrine, norepinephrine, growth hormone, glucagon, and several cytokines [1]. The typical clinical picture is hyperglycemia and insulin resistance. Acute stress-induced hyperglycemia is observed in many clinical conditions, such as myocardial infarction, stroke, trauma, and in critically ill surgical patients [2-5]. The causal relationships between hyperglycemia, insulin treatment, and prognosis have been the focus of attention in recent years [6].

Increasing evidence shows that adipose tissue plays a key role in the development of metabolic syndrome (MS) via the secretion of a variety of adipokines [7]. These adipokines include adiponectin, leptin, resistin and visfatin, among others. Studies of these adipokines have revealed a well-established link between adipose tissue, inflammation, and insulin resistance [8]. Because many acute metabolic changes seen in critically ill patients are similar to those seen in patients with MS, it is believed that such changes are also involved in the acute stress responses associated with critical illness [9].

Adipocyte fatty-acid binding protein (A-FABP) is one of the most abundant intracellular lipid transport proteins in mature adipocytes and macrophages. The expression is regulated by fatty acids, peroxisome proliferator-activated receptor γ agonists, lipopolysaccharide (LPS), oxidized low density lipoprotein and advanced glycation end products [10]. A-FABP-deficient mice exhibited reduced hyperinsulinemia and insulin resistance in the context of both dietary and genetic obesity, which might be caused by decreased TNF-alpha expression, enhanced insulin receptor signaling and muscle AMP-activated kinase activity [11]. A-FABP has also been shown to regulate many inflammatory cytokines *in vivo *and *in vitro *[12]. However, data from both rodents and humans suggest that it is also secreted by adipose tissue into the bloodstream [13]. Recent studies have confirmed its roles in various conditions associated with insulin resistance, including MS, diabetes mellitus, nonalcoholic fatty liver disease, Cushing's syndrome, and polycystic ovary disease [14-18]. The aim of our study was to evaluate the correlations between A-FABP, systemic inflammation, and insulin resistance associated with critical illness, and further to investigate its role in outcome prediction.

## Materials and methods

### Study design

A total of 120 patients (74 male, median age 74 years) was prospectively enrolled at the time of medical ICU admission in 2011. Patients currently undergoing regular hemodialysis, with acute myocardial infarction, who refused intensive therapy, or who were expected to stay < 72 hours in the ICU (for example, for post-interventional observation, acute intoxication) were excluded from the study. Written informed consent was obtained from each participant or from his or her family member, and the study was approved by the Institutional Review Board of the Taoyuan General Hospital. Patient data and clinical information, including Acute Physiology and Chronic Health Evaluation II (APACHE II) score, vasopressor demand, ventilator use or not, and total ICU and hospital days, were collected from medical records. Blood samples were collected within 48 hours of ICU admission. Patients with critical illness were divided into two groups: sepsis patients and non-sepsis patients. Sepsis was defined as the presence of systemic inflammatory response syndrome associated with infection confirmed or strongly suspected, according to criteria proposed by the American College of Chest Physicians and the Society of Critical Care Medicine Consensus Conference Committee for severe sepsis and septic shock [19]. Insulin resistance was calculated by the homeostasis model assessment (HOMA) method. Serum A-FABP concentrations were analyzed by ELISA according to the manufacturer's instructions. The median length of stay in the ICU was 7 days, and the median length of stay in hospital was 15 days.

The control group consisted of 45 age- and gender-matched healthy subjects with normal blood counts, renal function, and C-reactive protein (CRP) levels who were recruited from a health examination center.

### Statistical analysis

Because of the skewed distribution of most parameters in patients with a critical illness, data are given as median and IQRs (Table 1). Differences between two groups were assessed by the Mann-Whitney U test. Correlations between variables were analyzed using Spearman's correlation coefficient. The prognostic value of the variables was tested by univariate and multivariate analyses using a Cox regression model. Kaplan-Meier curves were plotted to display the impact on survival. Analyses were performed using STATA statistical software (release 10.0, StataCorp LP, Texas, USA). All statistical tests were two-sided, and *P *values < 0.05 were considered statistically significant.

**Table 1:** Characteristics of patients with critical illness.

Parameter	All patients (number = 120)	Sepsis patients (number = 72)	Non-sepsis patients (number = 48)
Male, number (%)	74 (62%)	47 (63%)	27 (60%)
Age, median (y)ear	74 (58.5, 82.5)	76 (63, 83.5)	70.5 (52, 80.5)
BMI, median (m^2^/kg)	22.7 (20.2, 24.8)	21.6 (19.5, 23.8)	23.9 (21.7, 27.4)
Hypertension, number (%)	58 (48%)	37 (51%)	21 (44%)
Preexisting diabetes, number (%)	46 (38%)	30 (42%)	16 (33%)
Smoking, number (%)	55 (46%)	35 (49%)	20 (42%)
Cardiovascular disease, number (%)	37 (31%)	21 (29%)	16 (33%)
APACHE II score, median	18 (13, 25)	20.5 (16, 26)	14 (12, 22)
ICU days, median	7 (3, 14)	7 (4, 14.5)	6 (3, 13)
Hospital days, median	15 (9, 26)	18.5 (10, 30)	13 (8, 20)
Death during ICU, number (%)	14 (11.7%)	13 (17.3%)	1 (2.2%)
Death before discharge, number (%)	20 (16.7%)	17 (22.7%)	3 (6.7%)
Mechanical ventilation, number (%)	62 (51.7%)	38 (50.7%)	24 (53.3%)
Ventilation time, median (days)	10 (6.5, 24.5)	10.5 (8, 22.5)	9.5 (5, 35)
Fasting glucose, median (mg/dl)	165 (115, 234)	171 (117, 234)	159 (115, 217)
Insulin, median (uIU/ml)	16 (10.8, 22.7)	18.1 (10.8, 23.1)	12.4 (9.1, 22.5)
Creatinine, median (mg/dl)	1.5 (1, 2.25)	1.7 (1.1, 2.6)	1.2 (0.9, 1.8)
Albumin, median (g/dl)	2.9 (2.5, 3.3)	2.7 (2.4, 3.1)	3.3 (2.8, 3.7)
Alkaline phosphatase, median (mg/dl)	26 (17, 41)	25 (16.5, 40)	26.5 (17.5, 42)
C-reactive protein, median (mg/dl)	10.55 (3.38, 23)	13.4 (7.59, 25.8)	5.36 (1.17, 8.08)
TNF-alpha, median (pg/ml)	3.44 (2.08, 7.73)	4.76 (2.57, 9.84)	2.77 (1.50, 3.92)
IL-6, median (pg/ml)	8.89 (6.69, 22.58)	7.87 (6.61, 24.79)	9.80 (7.14, 19.25)
Lactic acid, median (mmol/l)	3.3 (2.1, 5.9)	3.6 (2.5, 6.2)	2.5 (1.5, 5.4)
Procalcitonin, median (ug/l)	1.53 (0.25, 9.90)	4.04 (0.54, 14.03)	0.57 (0.10, 2.06)
A-FABP, median (ng/ml)	43.3 (25.7, 67.2)	46.2 (31.9, 70.2)	38.5 (17.1, 64.3)

A-FABP, adipocyte fatty-acid binding protein; APACHE, Acute Physiology and Chronic Health Evaluation; BMI, body mass index.

## Results

Among the 120 critically ill patients enrolled in this study, 72 met the criteria for sepsis (Table 1). In the majority of sepsis patients, the origin of infection was identified as pneumonia, urinary tract infection or intra-abdominal infection (Table 2). Non-sepsis patients did not differ from sepsis patients in terms of age or sex, and were admitted due to cardiopulmonary disorders (chronic obstructive pulmonary disease, asthma and cardiac pulmonary edema), cerebrovascular disease, decompensated liver disease, or other critical conditions. Among all critically ill patients, 11.7% died in the ICU and 16.7% died before discharge. Patients with sepsis clearly had a higher ICU and hospital mortality rate than non-sepsis patients (*P *= 0.008 and 0.01, respectively).

**Table 2:** Disease etiology of the study population.

	Sepsis patients (number = 72)	Non-sepsis patients (number = 48)
Etiology of sepsis critical illness (site of infection)		
Pulmonary	39	
Urinary	19	
Abdominal	10	
Others	4	
Etiology of non-sepsis critical illness		
Decompensated liver disease		10
Cardiovascular disease		16
Chronic obstructive pulmonary disease/asthma		9
Others		13

Compared with healthy volunteers, patients with critical illness had higher serum A-FABP levels (median 43.3 ng/ml in patients versus 19.3 ng/ml in controls; *P *< 0.0001). Subgroup analysis of septic and non-septic patients showed a trend of higher A-FABP serum levels in the septic patients (*P *= 0.08), especially in those with septic shock (median 53.25 ng/ml versus 35.13 ng/ml in non-shock patients, *P *= 0.004). Previous studies of MS and atherosclerosis found that women have higher circulating A-FABP levels. In this cohort of critically ill patients, there was still a trend for a gender difference in A-FABP concentrations (median 53.3 ng/ml in women versus 39.1 ng/ml in men, *P *= 0.057). Another important point that needs to be mentioned is that pre-existing diabetes patients did not have higher A-FABP concentrations than non-diabetic patients (median 43.6 ng/ml in diabetes versus 40.9 ng/ml in non-diabetes, *P *= 0.74), and there was no significant association between body mass index (BMI) and A-FABP (rho = -0.02, *P *= 0.83; Table 3). In subgroup analysis based on disease etiologies, there was no significant difference in serum A-FABP concentration within different disease etiologies (data not shown).

**Table 3:** Univariate and multivariate analyses of A-FABP concentrations with other parameters in all patients with critical illness.

	Univariate		Multivariate	
	
	rho	*P*	rho	*P*
Age	0.24	0.0085	0.11	0.41
BMI	-0.02	0.83		
Creatinine	0.42	< 0.0001	0.40	0.001
Fasting glucose	0.28	0.0025	0.31	0.04
HOMA-IR	0.24	0.038		
Total cholesterol	-0.28	0.0096	-0.26	0.04
Triglyceride	0.16	0.14		
CRP	0.10	0.43		
TNF-alpha	0.47	< 0.0001	0.51	< 0.0001
IL-6	-0.10	0.286		
Procalcitonin	0.26	0.004	0.25	0.05
Lactic acid	0.29	0.002	0.17	0.18
Albumin	-0.30	0.0017	-0.34	0.007
APACHE II score	0.42	< 0.0001	0.31	0.016

Rho and *P *value by Spearman rank correlation. Variables included in multivariate analysis are age, creatinine, fasting glucose, total cholesterol, TNF-alpha, procalcitonin, lactic acid, albumin, and APACHE II score. A-FABP, adipocyte fatty-acid binding protein; APACHE II, Acute Physiology and Chronic Health Evaluation; BMI, body mass index; CRP, C-reactive protein; HOMA-IR, homeostasis model assessment index of insulin resistance.

The correlations between circulating A-FABP and other well-known parameters are shown in Table 3. These parameters included markers of inflammation, sepsis, organ dysfunction, tissue perfusion and metabolism. Serum A-FABP was correlated positively with procalcitonin (rho = 0.26, *P *= 0.004) and lactic acid (rho = 0.29, *P *= 0.002), markers of bacterial infection and tissue hypoperfusion. With respect to inflammatory biomarkers, A-FABP was correlated with TNF-alpha (rho = 0.47, *P *< 0.0001), but not IL-6 and CRP. Renal failure was associated with elevated serum A-FABP, as A-FABP was correlated with creatinine (rho = 0.42, *P *< 0.0001). Albumin, a marker of hepatic biosynthetic capacity, was negatively correlated with A-FABP level (rho = -0.30, *P *= 0.0017). For the total patient cohort, A-FABP level was correlated with insulin resistance, as calculated by the HOMA-IR (rho = 0.24, *P *= 0.048) and fasting glucose (rho = 0.28, *P *= 0.0025). Cholesterol, a marker of lipid metabolism, was found to be inversely correlated with serum A-FABP (rho = -0.28, *P *= 0.0096). Most importantly, A-FABP was significantly correlated with the APACHE II score (rho = 0.42, P < 0.0001), a well-known scoring system in disease severity grading. After multivariate analysis, serum creatinine, fasting glucose, total cholesterol, TNF-alpha, albumin, and APACHE II score were still independently associated with A-FABP.

**Figure 1:**
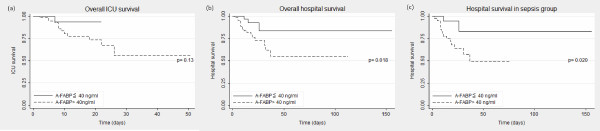
**Prognostic relevance of serum A-FABP concentrations in critically ill patients presented by Kaplan-Meier survival curves**. In ICU mortality analysis, the short-term mortality difference is not statistically significant between patients with higher and lower A-FABP concentrations (A-FABP cutoff value 40 ng/ml) (**a**). However, higher A-FABP levels do predict worse overall hospital outcome (**b**), especially in patients with sepsis (**c**). *P *values from Cox regression analysis are given. A-AFBP, adipocyte fatty-acid binding protein.

Cox regression analyses and Kaplan-Meier curves were used to assess the association of A-FABP with ICU and hospital survival (Figure 1). We identified a lower A-FABP level upon admission to the ICU as a prognostic marker for hospital survival in critically ill patients (hazard ratio (HR) 3.92, 95% CI 1.15 to 13.40, *P *= 0.029), especially in the sepsis patient group (HR 4.82, 95% CI 1.10 to 21.10, *P *= 0.037). Kaplan-Meier curves, using a cutoff value for serum A-FABP of 40 ng/ml, revealed significantly improved hospital survival for critically ill patients with low A-FABP levels upon admission to the ICU. We next tested whether A-FABP was an independent prognostic indicator of mortality in the group of critically ill patients with sepsis. Multivariate Cox regression analyses using A-FABP, APACHE II score and lactic acid as discriminators, revealed that a higher serum A-FABP level was still an independent predictor of worse hospital survival (HR 4.59, 95% CI 1.02 to 20.6, *P *= 0.047).

## Discussion

Our findings demonstrate that elevated serum A-FABP concentrations in patients with critical illness were positively correlated with APACHE II scores, inflammatory cytokine TNF-alpha and insulin resistance. Moreover, higher A-FABP concentrations independently predicted worse hospital outcome in patients with sepsis. Several adipokines, including adiponectin, retinol binding protein 4 (RBP4), resistin and leptin, were studied in critically ill patients by Koch *et al*. [20-23]. However, these results were inconsistent. Serum RBP4 and resistin concentrations in critically ill patients were different from normal controls, but adiponectin and leptin concentrations were not. The inflammatory biomarkers were correlated with RBP4, resistin and leptin concentrations, but not with adiponectin. The only common factor among these adipokines was that they were all associated with glucose or HOMA-IR, which reflects their well-known and dominant roles in insulin resistance.

In recent studies, A-FABP was found to be expressed in the ovary, spleen, bronchial epithelial cells, endothelial cells and keratinocytes, as well as some tumor cells [24]. Because adipose tissue is considered to be the main source of circulating A-FABP, we believe that upregulation of A-FABP expression and/or increased secretion from the adipose tissue contributes to elevated levels in patients with critical illness. It is supported by Jernas' study, which demonstrates that adipose tissue gene expression changes in patients with critical illness [25]. Another study by Han also showed that intermittent hypoxia induced A-FABP expression in endothelial cells [26]. The mechanism by which cytoplasmic A-FABP is released into circulation is also an unresolved question. Nevertheless, other FABPs in the serum are considered to be biochemical markers of tissue injury in related cells that produce FABP proteins: heart FABP for acute myocardial infarction, brain FABP for brain injury, intestinal FABP for intestinal damage [27,28]. In sepsis, elevated serum heart FABP and urinary liver FABP levels are believed to indicate myocardial and kidney injury as well [29,30]. Since the A-FABP level is higher in septic shock patients and positively correlated with lactic acid, the elevated A-FABP in critical illness might also reflect hypoperfusion and injuries to adipose tissues. The upregulation of A-FABP might impair nitric oxide production, suppress cardiomyocyte contraction and further deteriorate the hemodynamics [31,32]. However, it is well established that serum A-FABP is increased in patients with chronic renal failure, making renal clearance a relevant confounding factor for serum A-FABP concentrations. In our multivariate analysis, creatinine was independently correlated with serum A-FABP, indicating that impaired renal excretion is an important mechanism regulating A-FABP.

Our results also showed a positive correlation between serum A-FABP and fasting glucose and HOMA-IR, which has also been found in patients with MS. Indeed, data from basic and clinical studies have proved that circulating A-FABP is not only a biomarker of MS but is also involved in the pathogenesis of insulin resistance. The A-FABP concentrations in our study were not associated with pre-existing diabetes and BMI. These findings are in agreement with our theory that circulating A-FABP reflects critical illness-related insulin resistance. The development of insulin resistance in critical illness seems to be an adaptive response to infection or stress, whereby energy reserves are directed to where they are most needed, namely, the immune response. Interestingly, our data demonstrate a positive correlation of circulating A-FABP with TNF-alpha and procalcitonin, and we believe that bacterial infection might be a more important contributor than stress alone. In fact, inflammatory cytokines (for example, TNF-alpha) and Toll-like receptor 4 (TLR4), an obligatory receptor for bacterial LPS, are important participants in insulin resistance in critical illness. After free fatty acid and LPS binding to TLR4, A-FABP is known to be up-regulated in adipocytes and macrophages, which further increases TNF-alpha expression [33]. This offers another hint that A-FABP might be an important mediator between inflammation and insulin resistance in sepsis. Since statin treatment is known to decrease serum A-FABP levels in patients with hyperlipidemia [34], it is of interest to evaluate the possible benefits of statin treatment in sepsis through A-FABP regulation.

There are several limitations to our study. First, the observational study design and cross sectional data failed to clarify the causal relationship of A-FABP and sepsis. Administration of specific A-FABP antagonists in the animal model of sepsis may be helpful to answer this question. Second, there is an obvious ICU mortality difference between that found in routine clinical practice (around 20%) and that observed in our patients. This is because patients in the most serious condition were more likely to refuse to join our study. Some even refused invasive treatment and cardiopulmonary resuscitation. We also failed to obtain blood samples from patients who died on the day of ICU admission. Therefore, further studies are required to confirm our findings and to extend the conclusions to all critically ill patients. Third, all of these critically ill patients received suitable treatment from the emergency department, including antibiotics, fluid resuscitation, and inotropic agents. Most blood samples were collected around 12 to 48 hours after ICU admission. It is thus impossible to neglect the impact of different therapeutic agents on serum A-FABP concentrations.

## Conclusions

Our study has demonstrated that critically ill patients have higher serum A-FABP concentrations, which are correlated with markers of sepsis, renal function, tissue perfusion and insulin resistance. We offer evidence that A-FABP might act as a link between acute inflammation and altered metabolic homeostasis. Because higher A-FABP within 48 hours of ICU admission independently predicts a worse hospital outcome, it serves as a novel biomarker for all critically ill patients.

## Key messages

• Adipocyte fatty-acid binding protein (A-FABP), one of the most abundant intracellular lipid transport proteins in mature adipocytes and macrophages, was established as a link between inflammation and insulin resistance.

• A-FABP serum concentrations are elevated in critically ill patients, and are correlated with disease severity.

• In critical illness, A-FABP is associated with fasting glucose and HOMA-IR, but not with pre-existing diabetes or obesity.

• A-FABP correlates with TNF-alpha, procalcitonin and lactic acid, but not CRP.

• A-FABP is an independent prognostic factor for hospital survival in critically ill patients with sepsis.

## Abbreviations

A-FABP: adipocyte fatty-acid binding protein; APACHE: Acute Physiology and Chronic Health Evaluation; BMI: body mass index; CRP: C-reactive protein; ELISA: enzyme-linked immunosorbent assay; HOMA-IR: homeostasis model assessment index of insulin resistance; HR: hazard ratio; IL: interleukin; LPS: lipopolysaccharide; MS: metabolic syndrome; RBP4: retinol binding protein 4; TLR4: toll-like receptor 4; TNF: tumor necrosis factor.

## Competing interests

The authors declare that they have no competing interests.

## Authors' contributions

CLH, YWW, WJC, and WSY designed the study, and analyzed and interpreted the data. YHH performed the A-FABP measurements. ARH performed the statistical analysis. CLH and WSY wrote the manuscript. All authors have read and approved the manuscript for publication.

## References

[B1] Losser MR Damoisel C Payen D Bench-to bedside review: glucose and stress conditions in the intensive care units Crit Care 2010 17 231 10.1186/cc9100 20727232 PMC2945096

[B2] Capes SE Hunt D Malmberg K Gerstein HC Stress hyperglycaemia and increased risk of death after myocardial infarction in patients with and without diabetes: a systematic overview Lancet 2000 17 773 778 10.1016/S0140-6736(99)08415-9 10711923

[B3] Laird AM Miller PR Kilgo PD Meredith JW Chang MC Relationship of early hyperglycemia to mortality in trauma patients J Trauma 2004 17 1058 1062 10.1097/01.TA.0000123267.39011.9F 15179246

[B4] Capes SE Hunt D Malmberg K Pathak P Gerstein HC Stress hyperglycemia and prognosis of stroke in nondiabetic and diabetic patients: a systematic overview Stroke 2001 17 2426 2432 10.1161/hs1001.096194 11588337

[B5] Mowery NT May AK Collier BC Dossett LA Gunter OL Dortch MJ Diaz JJ Jr Glucose metabolism, not obesity, predicts mortality in critically ill surgical patients Am Surg 2010 17 1377 1383 21265352

[B6] Krinsley J Preiser JC Intensive insulin therapy to control hyperglycemia in the critically ill: a look back at the evidence shapes the challenges ahead Crit Care 2010 17 330 10.1186/cc9275 21143774 PMC3220034

[B7] Deng Y Scherer PE Adipokines as novel biomarkers and regulators of the metabolic syndrome Ann N Y Acad Sci 2010 17 E1 19 21276002 10.1111/j.1749-6632.2010.05875.x PMC3075414

[B8] Fève B Bastard JP Vidal H Relationship between obesity, inflammation and insulin resistance: new concepts C R Biol 2006 17 587 597 Article in French 10.1016/j.crvi.2006.03.020 16860277

[B9] Andreelli F Jacquier D Troy S Molecular aspects of insulin therapy in critically ill patients Curr Opin Clin Nutr Metab Care 2006 17 124 130 10.1097/01.mco.0000214571.97933.0a 16477177

[B10] Furuhashi M Hotamisligil GS Fatty acid-binding proteins: role in metabolic diseases and potential as drug targets Nat Rev Drug Discov 2008 17 489 503 10.1038/nrd2589 18511927 PMC2821027

[B11] Furuhashi M Fucho R Görgün CZ Tuncman G Cao H Hotamisligil GS Adipocyte/macrophage fatty acid-binding proteins contribute to metabolic deterioration through actions in both macrophages and adipocytes in mice J Clin Invest 2008 17 2640 2650 18551191 10.1172/JCI34750 PMC2423863

[B12] Hui X Li H Zhou Z Lam KS Xiao Y Wu D Ding K Wang Y Vanhoutte PM Xu A Adipocyte fatty acid-binding protein modulates inflammatory responses in macrophages through a positive feedback loop involving c-Jun NH2-terminal kinases and activator protein-1 J Biol Chem 2010 17 10273 10280 10.1074/jbc.M109.097907 20145251 PMC2856232

[B13] Xu A Wang Y Xu JY Stejskal D Tam S Zhang J Wat NM Wong WK Lam KS Adipocyte fatty acid binding protein is a plasma biomarker closely associated with obesity and metabolic syndrome Clin Chem 2006 17 405 413 10.1373/clinchem.2005.062463 16423904

[B14] Xu A Tso AW Cheung BM Wang Y Wat NM Fong CH Yeung DC Janus ED Sham PC Lam KS Circulating adipocyte-fatty acid binding protein levels predict the development of the metabolic syndrome: a 5-year prospective study Circulation 2007 17 1537 1543 10.1161/CIRCULATIONAHA.106.647503 17389279

[B15] Toruner F Altinova AE Akturk M Kaya M Arslan E Bukan N Kan E Yetkin I Arslan M The relationship between adipocyte fatty acid binding protein-4, retinol binding protein-4 levels and early diabetic nephropathy in patients with type 2 diabetes Diabetes Res Clin Pract 2011 17 203 207 10.1016/j.diabres.2010.11.011 21176857

[B16] Kim YC Cho YK Lee WY Kim HJ Park JH Park DI Sohn CI Jeon WK Kim BI Park SE Rhee EJ Park CY Oh KW Park SW Kim SW Ryu SH Serum adipocyte-specific fatty acid-binding protein is associated with nonalcoholic fatty liver disease in apparently healthy subjects J Nutr Biochem 2011 17 289 292 10.1016/j.jnutbio.2010.02.007 20579864

[B17] Durovcová V Marek J Hána V Matoulek M Zikán V Haluzíková D Kaválková P Lacinová Z Kršek M Haluzík M Plasma concentrations of adipocyte fatty acid binding protein in patients with Cushing's syndrome Physiol Res 2010 17 963 971 20533865 10.33549/physiolres.931842

[B18] Wang J Tang J Wang B Song J Liu J Wei Z Zhang F Ma X Cao Y FABP4: a novel candidate gene for polycystic ovary syndrome Endocrine 2009 17 392 396 10.1007/s12020-009-9228-5 19844814

[B19] Bone RC Balk RA Cerra FB Dellinger RP Fein AM Knaus WA Schein RM Sibbald WJ Definitions for sepsis and organ failure and guidelines for the use of innovative therapies in sepsis. The ACCP/SCCM Consensus Conference Committee. American College of Chest Physicians/Society of Critical Care Medicine Chest 1992 17 1644 1655 10.1378/chest.101.6.1644 1303622

[B20] Koch A Weiskirchen R Sanson E Zimmermann HW Voigt S Dückers H Trautwein C Tacke F Circulating retinol binding protein 4 in critically ill patients before specific treatment: prognostic impact and correlation with organ function, metabolism and inflammation Crit Care 2010 17 R179 10.1186/cc9285 20932285 PMC3219283

[B21] Koch A Gressner OA Sanson E Tacke F Trautwein C Serum resistin levels in critically ill patients are associated with inflammation, organ dysfunction and metabolism and may predict survival of non-septic patients Crit Care 2009 17 R95 10.1186/cc7925 19545363 PMC2717467

[B22] Koch A Weiskirchen R Zimmermann HW Sanson E Trautwein C Tacke F Relevance of serum leptin and leptin-receptor concentrations in critically ill patients Mediators Inflamm 2010 17 pii: 473540 10.1155/2010/473540 PMC2943118 20871818

[B23] Koch A Sanson E Voigt S Helm A Trautwein C Tacke F Serum adiponectin upon admission to the intensive care unit may predict mortality in critically ill patients J Crit Care 2011 17 166 174 10.1016/j.jcrc.2010.07.015 20869198

[B24] Furuhashi M Ishimura S Ota H Miura T Lipid chaperones and metabolic inflammation Int J Inflam 2011 17 642612 22121495 10.4061/2011/642612 PMC3206330

[B25] Jernås M Olsson B Sjöholm K Sjögren A Rudemo M Nellgård B Carlsson LM Sjöström CD Changes in adipose tissue gene expression and plasma levels of adipokines and acute-phase proteins in patients with critical illness Metabolism 2009 17 102 108 10.1016/j.metabol.2008.08.012 19059537

[B26] Han Q Yeung SC Ip MS Mak JC Effects of intermittent hypoxia on A-/E-FABP expression in human aortic endothelial cells Int J Cardiol 2010 17 396 398 10.1016/j.ijcard.2010.04.027 20452069

[B27] Tanaka T Hirota Y Sohmiya K Nishimura S Kawamura K Serum and urinary human heart fatty acid-binding protein in acute myocardial infarction Clin Biochem 1991 17 195 201 10.1016/0009-9120(91)90571-U 2040092

[B28] Pelsers MM Hermens WT Glatz JF Fatty acid-binding proteins as plasma markers of tissue injury Clin Chim Acta 2005 17 15 35 10.1016/j.cccn.2004.09.001 15653098

[B29] Zhang ZC Dai HW Yu YH Yang JD Hu CB Usefulness of heart-type fatty acid-binding protein in patients with severe sepsis J Crit Care 2012 17 415 e13-8 22386224 10.1016/j.jcrc.2012.01.004

[B30] Doi K Noiri E Maeda-Mamiya R Ishii T Negishi K Hamasaki Y Fujita T Yahagi N Koide H Sugaya T Nakamura T Urinary L-type fatty acid-binding protein as a new biomarker of sepsis complicated with acute kidney injury Crit Care Med 2010 17 2037 2042 20657273 10.1097/CCM.0b013e3181eedac0

[B31] Aragonès G Saavedra P Heras M Cabré A Girona J Masana L Fatty acid-binding protein 4 impairs the insulin-dependent nitric oxide pathway in vascular endothelial cells Cardiovasc Diabetol 2012 17 72 10.1186/1475-2840-11-72 22709426 PMC3503556

[B32] Lamounier-Zepter V Look C Alvarez J Christ T Ravens U Schunck WH Ehrhart-Bornstein M Bornstein SR Morano I Adipocyte fatty acid-binding protein suppresses cardiomyocyte contraction: a new link between obesity and heart disease Circ Res 2009 17 326 334 10.1161/CIRCRESAHA.109.200501 19608978

[B33] Kazemi MR McDonald CM Shigenaga JK Grunfeld C Feingold KR Adipocyte fatty acid-binding protein expression and lipid accumulation are increased during activation of murine macrophages by toll-like receptor agonists Arterioscler Thromb Vasc Biol 2005 17 1220 1224 10.1161/01.ATV.0000159163.52632.1b 15705927

[B34] Wu YW Kao HL Huang CL Chen MF Lin LY Wang YC Lin YH Lin HJ Tzen KY Yen RF Chi YC Huang PJ Yang WS The effects of 3-month atorvastatin therapy on arterial inflammation, calcification, abdominal adipose tissue and circulating biomarkers Eur J Nucl Med Mol Imaging 2012 17 399 407 10.1007/s00259-011-1994-7 22109668

